# Supplementary material for: HIVprotI: an integrated web based platform for prediction and design of HIV proteins inhibitors
Source: J Cheminform. 2018 Mar 9;10:12. doi: 10.1186/s13321-018-0266-y (PMC5845081; doi:10.1186/s13321-018-0266-y)
Supplement: Supplementary file 2 — Additional file 2. Source code of HIVProtI web server. [file 13321_2018_266_MOESM2_ESM.zip › HIVprotI_Source-code/hivprotI/weka-3-6-6/doc/weka/core/stemmers/package-summary.html]

weka.core.stemmers


---


|  |  |  |
| --- | --- | --- |
| **Overview** \| **Package** \| Class \| **Tree** \| **Deprecated** \| **Index** \| **Help** \| **Weka's home** | |  |
| **PREV PACKAGE**   **NEXT PACKAGE** | **FRAMES**    **NO FRAMES**     **All Classes** |  |


---

## Package weka.core.stemmers

| **Interface Summary** | |
| --- | --- |
| **Stemmer** | Interface for all stemming algorithms. |

| **Class Summary** | |
| --- | --- |
| **IteratedLovinsStemmer** | An iterated version of the Lovins stemmer. |
| **LovinsStemmer** | A stemmer based on the Lovins stemmer, described here:    Julie Beth Lovins (1968). |
| **NullStemmer** | A dummy stemmer that performs no stemming at all. |
| **SnowballStemmer** | A wrapper class for the Snowball stemmers. |
| **Stemming** | A helper class for using the stemmers. |

---


|  |  |  |
| --- | --- | --- |
| **Overview** \| **Package** \| Class \| **Tree** \| **Deprecated** \| **Index** \| **Help** \| **Weka's home** | |  |
| **PREV PACKAGE**   **NEXT PACKAGE** | **FRAMES**    **NO FRAMES**     **All Classes** |  |


---
